# Syringomatous Tumour Presenting as Inversion of a Supernumerary Nipple

**DOI:** 10.1155/2019/9461815

**Published:** 2019-01-17

**Authors:** Louise Öwall, Henrik Mygind, Åsa Rosenborg, Anne-Vibeke Lænkholm

**Affiliations:** ^1^Department of Plastic and Breast Surgery, Zealand University Hospital, Roskilde, Denmark; ^2^Department of Pathology, Zealand University Hospital, Slagelse, Denmark; ^3^Department of Radiology, Zealand University Hospital, Ringsted, Denmark

## Abstract

Syringomatous tumour (SyT) is a rare type of benign locally infiltrative tumour with debated origin. Because of the growth pattern, SyT can be mistaken for a malignant tumour, and it is therefore important to keep this diagnosis in mind. This case presents a woman with two supernumerary nipples on each side of the abdomen. One of the nipples was inverted with a small palpable firm mass in close relation to the nipple, leading to referral to the breast surgery department. SyT occurring in a supernumerary nipple and presenting with the symptoms described in this case has to our knowledge never been described previously.

## 1. Introduction

Syringomatous tumour of the nipple (SyT) is a rare locally infiltrative tumour of the nipple, which histologically resembles skin adnexal tumours [1, 2]. The origin of SyT is still debatable with reports suggesting different sites of origin, including eccrine sweat gland ducts of the skin within the area of the nipple, [3] pluripotent adnexal keratinous cysts, and [4] distinct breast epithelium progenitor cells with the capacity of both squamous and glandular differentiation [5, 6]. SyT is not known to metastasize, but due to the locally infiltrative and diffuse growth pattern there is a risk for local recurrence. The recommended treatment is resection with generous margins. SyT can present with symptoms such as a firm palpable mass in relation to the nipple, discharge, or inversion of the nipple [1, 2]. In general, SyT has previously been described in association to the nipple of the normal breast [7–11]. Only two previously published cases describe SyT in supernumerary breast tissue: a woman with SyT in supernumerary breast tissue that became symptomatic due to physiological changes during pregnancy [12] and one other case describing a young girl who had a supernumerary nipple removed for cosmetic reasons, where SyT was an accidental finding during microscopic examination [13].

This case presents a woman with two supernumerary nipples on each side of the abdomen. One of the nipples was inverted with a small palpable firm mass in close relation to the nipple, leading to referral to the breast surgery department.

## 2. Case Presentation

**Figure 1:**
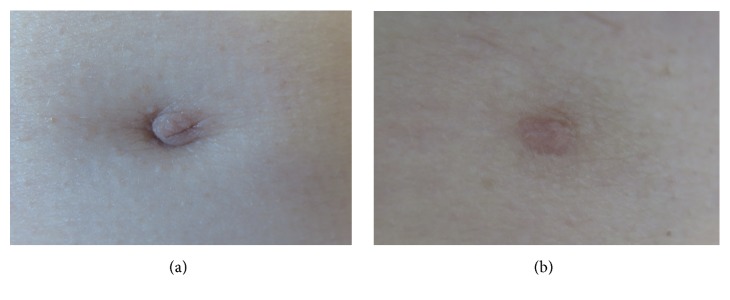
Clinical photo of the supernumerary nipple on the right (a) and left side (b) of the upper abdominal wall. The one on the right side (a) presents with visible inversion of the supernumerary nipple.

**Figure 2:**
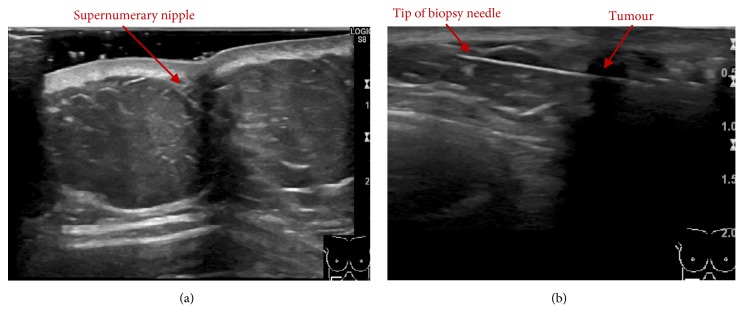
Ultrasound of upper abdominal wall right side (a). Ultrasound guided needle core biopsy (16G) of the tumour located on the upper abdominal wall right side (b).

A 61-year-old woman presented with two supernumerary nipples located along the milk line on each side of the upper abdomen. During a few months before referral, the patient had noticed a firm palpable mass in close relations to the supernumerary nipple on the right side (Figure 1). She had no other symptoms. Bilateral mammogram and ultrasound revealed normal breast parenchyma. Ultrasound of the supernumerary nipple on the right side confirmed a small mass in relation to this nipple, presenting as a hypoechoic, well-defined area, measuring approximately 10 mm in diameter (Figure 2).

**Figure 3:**
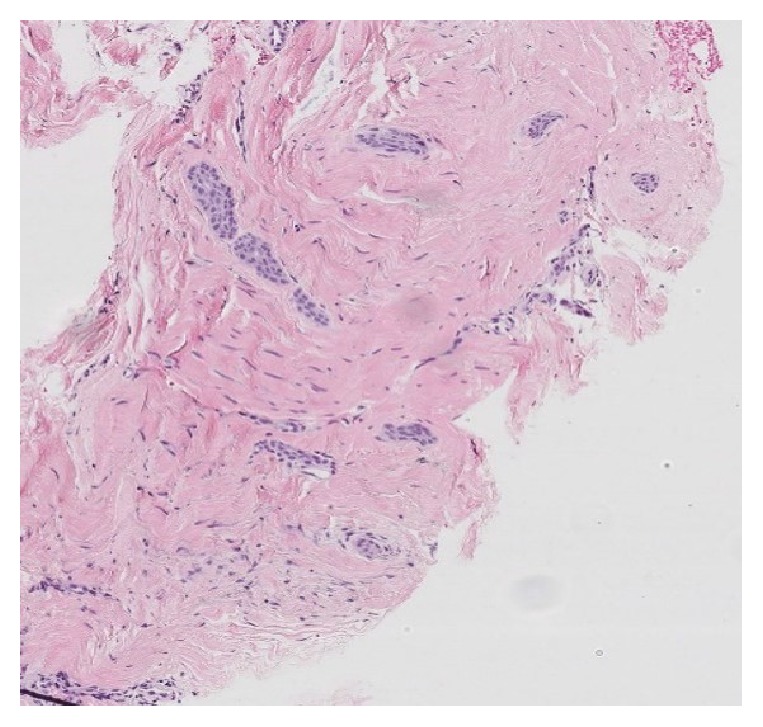
Micrograph of the needle core biopsy, presenting elongated epithelial islands composed of cubic cells with a small centrally located lumen of cuticles, surrounded by fibrous tissue.

Upon clinical examination, the supernumerary nipple on the right side presented with inversion and a palpable firm mass underneath this nipple. Ultrasound-guided needle core biopsy (16G) was performed (Figure 2(b)). The pathology report described elongated epithelial islands composed of cubic cells, with a small centrally located lumen with focally identifiable cuticles. The epithelial structures were surrounded by a dense fibrous stroma. The microscopic analysis indicated the possibility of SyT and the lesion was categorized as a B3-lesion (a lesion with uncertain malignant potential) (Figure 3).

**Figure 4:**
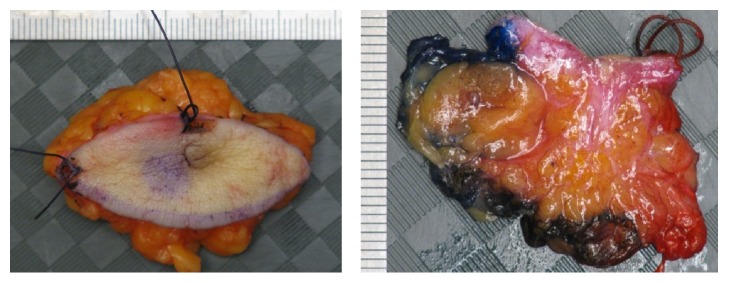
Excised supernumerary nipple specimen marked as a lumpectomy.

Following a Multidisciplinary Team Conference, it was decided to recommend resection with a 5 mm rim of normal tissue, which was subsequently performed (Figure 4).

**Figure 5:**
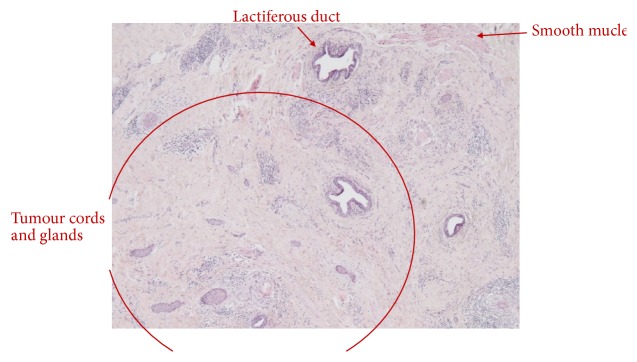
Micrograph of the tumour. Tumour cords and glands surrounding lactiferous ducts and smooth muscle.

Macroscopically the tumour measured 7 x 5 x 5 mm and was described as a firm and grey-white tumour, in close relations to the supernumerary nipple. Microscopically the tumour measured 13 mm in diameter and was localized in the deep part of dermis and underlying subcutaneous tissue with no relation to the epidermis. The tumour was composed of solid trabecular and glandular imitating formations with focal cysts. The epithelium consisted of cells with slightly irregular nuclei and indistinct nucleoli, surrounded by an eosinophilic cytoplasm. The glandular structures were lined by cuboidal cells. The tumour was in close association with the lactiferous ducts and smooth muscle of the nipple (Figure 5). Foreign body giant cell reactions, due to ruptured cysts, were identified.

**Figure 6:**
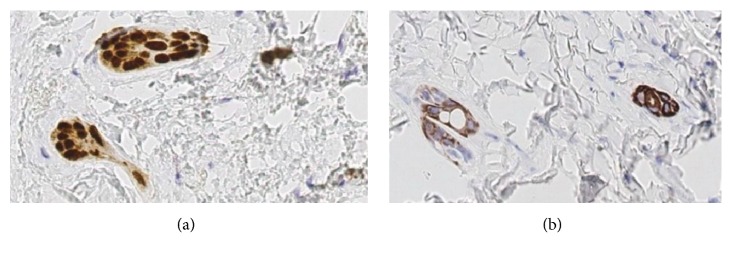
Micrograph of the immunohistochemical analysis showing positive reaction for P63 (a) and CK7 (b).

Immunohistochemical analysis showed positive reaction for CK5, CK14, and P63 (Figure 6(a)) in association with the epithelium presenting as solid cords, whereas the glandular luminal cells showed positive reaction for CK7 (Figure 6(b)). Thus, the immunohistochemical analysis demonstrated the complex nature of this lesion. The diagnosis was SyT based on the pathology report.

A re-excision was performed due to insufficient rim of normal tissue in the cranial direction in order to minimize the risk of recurrence.

## 3. Discussion

Due to the rarity of SyT, this type of tumour may constitute a diagnostic challenge. Primary malignant breast carcinoma as low-grade adenosquamous carcinoma (LGASC) and tubular carcinoma are mentioned as important differential diagnoses [1, 2]. However, in case of primary malignant breast carcinoma, ductal carcinoma in situ will often be present. Further, tubular carcinoma is most often estrogen receptor (ER) positive, whereas SyT is ER negative. A comparative study of LGASC and SyT showed high rates of* PIK3CA* mutations in LGASC as compared to SyT [14].

With respect to malignant skin adnexal tumours the most closely related tumours are syringoid carcinoma and microcystic adnexal carcinoma [15]. These tumours morphologically resemble SyT since they have features of both eccrine and follicular differentiation [16], but the presence of keratinous cysts and perineural growth pattern are more dominant in these lesions as compared to SyT. Syringoid carcinoma and microcystic adnexal carcinoma have the capacity to metastasize and follow-up is mandatory after local resection. In contrast, SyT has never been shown to metastasize [12]. Immunohistochemistry cannot be used to differentiate these lesions due to the potential common origin of the epithelial component.

Supernumerary nipples are not an uncommon abnormality, with a prevalence ranging from 1.7 to 3.75 % reported in the literature [17]. As described in this case, they are usually located along the milk line on the anterior chest wall. It is important to know that supernumerary nipples or ectopic breast tissue can be subjected to the same pathological lesions as the normal breast. Hence, pathological findings occurring in relation to a supernumerary nipple, or an area of ectopic breast tissue, should be examined just as thoroughly as any pathological findings occurring within the normal breast.

## 4. Conclusion

In conclusion, SyT is a rare type of “benign” locally infiltrative tumour with debated origin. Morphologically it can be difficult to differentiate it from malignant tumours. Only two previous cases in the literature describe this type of tumour occurring within supernumerary breast tissue or nipple. SyT occurring in a supernumerary nipple presenting with the symptoms described in this case has to our knowledge never been described previously.
